# Maternal and perinatal outcomes after vacuum-assisted vaginal delivery versus second-stage caesarean delivery in a generalist-run rural district hospital: A retrospective cohort study

**DOI:** 10.4102/safp.v68i1.6304

**Published:** 2026-08-25

**Authors:** Andrew G. Wilkins, James D. Porter, John-D K. Lotz, Olukayode Adeleke

**Affiliations:** 1 Department of Family Medicine and Rural Health, Faculty of Medicine and Health Sciences, Walter Sisulu University, Mthatha, South Africa; 2 Madwaleni Hospital, Eastern Cape Department of Health, Amathole District, Elliotdale, South Africa; 3 Department of Family, Community and Emergency Care, Faculty of Health Sciences, University of Cape Town, Cape Town, South Africa; 4 False Bay Hospital, Metro Health Services, Western Cape Department of Health and Wellness, Cape Town, South Africa

**Keywords:** vacuum-assisted delivery, vacuum extraction, caesarean section, second stage of labour, maternal and perinatal outcomes

## Abstract

**Background:**

Vacuum-assisted vaginal deliveries (VADs) are recommended as safe alternatives to second-stage caesarean deliveries (SSCDs); however, little is known about their use and associated outcomes in South African rural district hospitals without obstetric specialist support.

**Methods:**

A 5-year retrospective cohort study was conducted at a rural district hospital in South Africa. Women who underwent a VAD attempt or SSCD in the second stage of labour were compared with respect to baseline sociodemographic and clinical characteristics, and maternal and perinatal outcomes. Multivariable logistic regression was used to estimate adjusted odds ratios (aORs).

**Results:**

Of 7293 deliveries during the study period, 567 met inclusion criteria, including 417 VAD attempts and 150 SSCDs. Baseline characteristics were largely similar, with SSCDs more frequently performed in women with a previous caesarean delivery, for prolonged second stage of labour, and by community service medical officers. Vacuum-assisted delivery attempts were more commonly used for foetal distress and were associated with significantly shorter decision-to-delivery intervals (< 30 min in 79% vs 21%, *p* < 0.01). After adjustment, VAD attempts were associated with reduced odds of postpartum haemorrhage (aOR 0.30, 95% confidence interval [CI] 0.15–0.60), with no significant difference in composite adverse maternal outcomes (aOR 0.64, 95% CI 0.39–1.05) or composite adverse perinatal outcomes (aOR 0.82, 95% CI 0.42–1.60).

**Conclusion:**

In this rural district hospital setting, VAD attempts performed by doctors without obstetric specialist support were associated with improved short-term maternal outcomes and equivalent perinatal outcomes compared with SSCDs.

**Contribution:**

These findings support the safe use of VAD in rural settings and highlight the potential contribution of family physicians and family medicine registrars to second-stage obstetric decision-making.

## Introduction

Improving maternal and perinatal health is a global health priority and forms part of the World Health Organization’s sustainable development goals.^1^ The highest maternal and perinatal mortality rates occur in low-income and middle-income countries (LMICs), and mothers delivering in rural areas are at the highest risk for adverse outcomes.^1,2^ Complications during labour contribute significantly towards adverse maternal and perinatal outcomes, with obstetric haemorrhage, pregnancy-related sepsis and anaesthetic complications causing more than 35% of maternal deaths with primary obstetric cause in South Africa in 2023.^3,4^ Offering safe interventions during labour is an essential component of improving outcomes globally, but especially in LMICs like South Africa.

Vacuum extraction and caesarean section are obstetric interventions employed for maternal or foetal indications during the second stage of labour, which is defined as the period from complete cervical dilatation to the full delivery of the foetus.^5,6^ Either intervention carries risks and benefits, and the decision of procedure should be individualised, depending on the indication for intervention, facility and healthcare provider ability, decision-to-delivery interval (DDI) and medico-legal considerations.^5^

South Africa and many other LMIC countries have seen a steady rise in caesarean delivery (CD) rates.^7,8^ The reasons for this include medicolegal concerns, income generation, healthcare convenience, the perceived benefits of CDs over normal vaginal or instrumental deliveries, and a lack of equipment and training to perform instrumental deliveries.^3,8^ There are safety concerns with the unnecessary use of CD as they are associated with increased risk of haemorrhage and infection, prolonged hospital stays, and need for blood transfusions.^3,4,9^ Furthermore, long-term risks of a scarred uterus – such as hysterectomy, abnormal placentation, uterine rupture, haemorrhage, and postpartum infection in future pregnancies – are important considerations.^3,10^ These risks are particularly high in rural settings where challenges in access to healthcare mean many births happen outside of healthcare facilities, and complicated deliveries are being performed by non-specialist medical practitioners with limited training and experience.^11^ In resource-poor settings, access to vacuum-assisted deliveries (VADs) can potentially improve maternal and neonatal outcomes and prevent unnecessary CD. Despite these recommendations, the use of VAD has almost disappeared from obstetric practice in most LMICs, with remarkably low rates of use.^3,8^ There is a paucity of literature on the use of VADs in South Africa and other LMICs, and the available literature is mostly from tertiary centres with specialist obstetricians overseeing care.^12,13,14^

The aim of this study was to compare maternal and perinatal outcomes of VADs and second-stage caesarean deliveries (SSCDs) performed by medical doctors with no obstetric specialist support at a rural district hospital in South Africa.

## Research methods and design

### Study design

This retrospective cohort study utilised data obtained from hospital records of all women who underwent either an attempted VAD or a SSCD at Madwaleni Hospital, over a 5-year period from 01 January 2018 to 31 December 2022.

### Study setting

Madwaleni Hospital is a district hospital in a rural area of the Eastern Cape Province of South Africa. The hospital serves a population of approximately 70 000, majority isiXhosa-speaking black South Africans from the Mbhashe subdistrict and Amathole district.^15^ The maternity ward records approximately 1150 deliveries each year, while the neonatal unit admits an average of 18 babies per month. All VAD attempts and CDs are performed by medical doctors with no obstetric specialist support, and babies are managed by the same clinicians. There is, however, variation in knowledge and experience among doctors. Community service medical officers, junior and senior medical officers, family medicine registrars, and family physicians possess varying levels of experience regarding the performance of interventions during the second stage of labour. During the study period, the medical officers ranged in experience from post-community service to 9 years of district hospital experience. The unit has an operating theatre available 24 h a day and manages patients according to national district hospital-level guidelines. Complex cases are transferred to Nelson Mandela Academic Hospital, a tertiary-level facility 90 min away. However, due to poor road and transport infrastructure, staffing challenges, and overwhelming patient numbers, many high-risk patients are managed at the district hospital.

### Participants

Complete purposive sampling of the accessible population over the selected period was conducted. A new reliable record-keeping system as well as the formalisation of the decentralised family medicine training program over this time directed the decision to include cases from this period only. All women who delivered a singleton pregnancy, > 34 weeks’ gestation, cephalic presentation and were assisted by a VAD attempt (regardless of success) or SSCD were included. Exclusion criteria were women with multiple gestation, non-cephalic presentation, < 34 weeks’ gestation, and CD in the first stage of labour. Women with cardiotocographic evidence of foetal distress as well as those who had a failed VAD attempt and subsequent CD or normal vaginal delivery (NVD) were included in the analysis.

### Data collection process

Files for all women who underwent CD were retrieved and screened to find those who had a fully dilated cervix before the decision to intervene. The delivery room register was screened to identify and retrieve files for those women who underwent VAD. Women who underwent CD following a failed VAD were analysed within the VAD attempt group, in keeping with the predefined intention-to-treat approach. Predetermined sociodemographic and clinical data points were collected from the maternal care records. Neonatal admission records were accessed by cross-referencing information from the maternal care records indicating the need for admission with the neonatal admission registers. An experienced research assistant with a clinical background supported the researcher with data collection. Key sociodemographic and baseline characteristics, intervention details, and outcomes were collected from patient notes as guided by previous literature.^12,13,14,16^ The key maternal outcome of interest was the adverse maternal outcome composite, defined as the presence of any of the following: postpartum haemorrhage (PPH), need for blood transfusion, major perineal injury, need for transfer to a tertiary hospital, need for further surgery, uterine rupture, maternal death, and prolonged hospital stay (more than 5 days). The key perinatal outcome of interest was the adverse perinatal outcome composite, which was defined as: need for admission, need for transfer to a tertiary hospital, major birth trauma, neonatal seizures, low Apgar score, foetal death after decision to deliver made, or early neonatal death.

Composite maternal and perinatal outcomes were employed to enhance statistical power for uncommon but clinically important adverse events. Despite heterogeneity in severity, all components reflect outcomes associated with increased morbidity, resource utilisation, or need for escalation of care in a rural district hospital setting.

### Statistical analysis

A free, secure electronic data collection tool, Kobo Toolbox (Cambridge, MA, US), was used for data collection. The platform is password-protected and requires authenticated user log-in, with two-factor authentication to prevent unauthorised access.^17^ Once cleaned and de-identified, data were then transferred to Stata version 18.0 (StataCorp, College Station, TX, US) for statistical analysis.^18^ For categorical data, frequencies and proportions were described by *n* (%) unless otherwise specified. Two intervention groups (‘VAD attempts’ and ‘SSCDs’) were compared in terms of baseline characteristics and outcomes (defined as above). Bivariate analyses were performed to determine associations between baseline maternal and neonatal demographic and clinical characteristics. Univariate and bivariate analyses were performed to estimate crude and adjusted odds ratios (aOR) for maternal and perinatal outcomes based on delivery intervention. Multivariate logistic regression analyses were then performed to estimate aOR with 95% confidence intervals (CIs) for the key primary maternal and perinatal study outcomes as well as PPH and low Apgar scores. *P* < 0.05 was used to define statistical significance.

### Ethical considerations

Ethical clearance to conduct this study was obtained from the Walter Sisulu University Research Ethics Committee (No. 146/2022).

## Results

**FIGURE 1:**
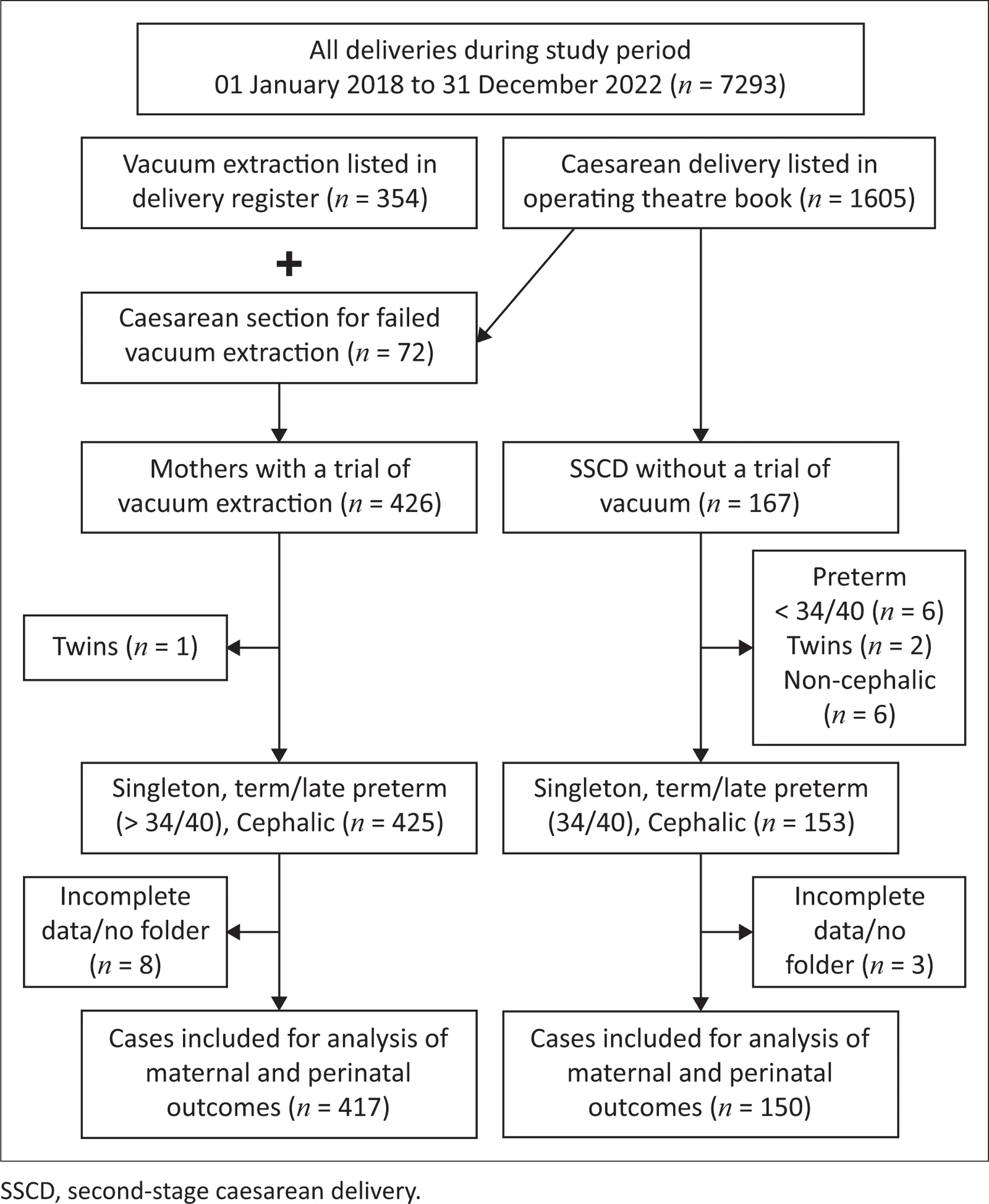
Flowchart of the study participation.

As shown in Figure 1, there was a total of 7293 deliveries during the study period, which included 354 vacuum extractions (4.8%), 1605 caesarean deliveries (22%), and 5334 normal vaginal deliveries (73.1%). There were 72 cases of failed vacuum extraction (16.9% failure rate) included in the VAD attempt cohort (intention a priori). A total of 567 cases met the inclusion criteria and were included in the analysis, consisting of the two cohorts of VAD (417) and SSCD without vacuum attempt (150).

Almost a third of participants were adolescents, two-thirds were nulliparous, and around a quarter were HIV-positive. There was a significant difference for previous CD, with 47 (11%) of the VAD attempt group and 29 (19%) of the SSCD group having had a previous CD (*p* = 0.01). Level of clinician for decision-making differed across groups with community service medical officers more likely to opt for a SSCD (39%) compared with VAD (21%); medical officers (junior and senior) more likely to opt for a VAD attempt (17% and 21%) compared with SSCD (10% and 14%); and family medicine registrars and family physicians equally as likely to opt for a VAD attempt (24% and 15%) or SSCD (23% and 15%) (*p* = 0.01). Decision-to-delivery interval was shorter in the VAD attempt group, with a median delivery time of 8 min compared with 49 min in the SSCD without vacuum attempt group (*p* < 0.01). Prolonged second stage was more likely the indication for intervention in the SSCD group (62%) compared with the VAD attempt group (51%), while foetal distress was more likely in the VAD attempt group (40%) compared with the SSCD group (33%) (*p* = 0.01). Other clinical and sociodemographic characteristics are shown in Table 1.

**TABLE 1:** Demographic and clinical characteristics.

Characteristics	All†		Intervention type†				*P*
			VAD attempt		SSCD		
	*n* (*N* = 567)	%	*n* (*N* = 417)	%	*n* (*N* = 150)	%	
**Mother’s age (years)**	-	-	-	-	-	-	0.19
< 20	165	29	120	29	45	30	-
20–34	350	62	264	63	86	57	-
≥ 35	52	9	33	8	19	13	-
**Number of antenatal care visits**	-	-	-	-	-	-	0.21
0	41	7	32	8	9	6	-
1–3	176	31	128	31	48	32	-
≥ 4	350	62	257	61	93	62	-
**Parity**	-	-	-	-	-	-	0.44
Nulliparous	377	67	283	68	94	63	-
Multiparous	177	31	124	30	53	35	-
Grand multiparous	13	2	10	2	3	2	-
**BMI** ‡	-	-	-	-	-	-	0.26
Underweight	20	4	14	3	6	4	-
Healthy	353	62	268	64	85	57	-
Overweight	194	34	135	33	59	39	-
**HIV status**	-	-	-	-	-	-	0.34
Positive	146	26	102	24	44	29	-
Negative	421	74	315	76	106	71	-
**Previous caesarean section**	-	-	-	-	-	-	**0.01**
Yes	77	14	47	11	29	19	-
No	490	86	370	89	121	81	-
**Maternal HPT disorder** §	-	-	-	-	-	-	0.12
Yes	57	10	37	9	20	13	-
No	510	90	380	91	130	87	-
**Level of clinician (decision)** ¶	-	-	-	-	-	-	**0.01**
Community service Medical Officer (MO)	145	26	87	21	58	39	-
MO < 2 years	84	15	69	17	15	10	-
MO ≥ 2 years	107	19	86	21	21	14	-
FM registrar	136	24	102	24	34	23	-
Family physician	84	15	62	15	22	15	-
Unknown	11	2	11	3	0	0	-
**Decision to delivery interval**	-	-	-	-	-	-	**< 0.01**
< 30 min	361	64	329	79	32	21	-
≥ 30 min	206	36	88	21	118	79	-
**GA at delivery**	-	-	-	-	-	-	0.53
< 37	39	7	31	7	8	6	-
37–40	253	45	191	46	62	41	-
> 40	275	48	195	47	80	53	-
**Birth weight**	-	-	-	-	-	-	0.76
< 2500 g	16	3	11	3	5	3	-
2500 g – 4000 g	522	92	386	92	136	91	-
> 4000 g	29	5	20	5	9	6	-
**Indication for intervention**	-	-	-	-	-	-	**0.01**
Prolonged second stage	306	54	213	51	93	62	-
Foetal distress	218	39	168	40	50	33	-
Maternal fatigue	25	4	24	6	1	1	-
Medical reason	3	0.5	2	0.5	1	1	-
Other	15	3	10	2	5	3	-
**Sex of newborn**	-	-	-	-	-	-	0.86
Male	316	56	230	55	86	57	-
Female	248	44	185	45	63	42	-
Unknown	3	0.5	2	0.5	1	0.5	-

Note: Bold values indicate statistically significant results (*p* < 0.05).

HIV, human immunodeficiency virus; HPT, hypertension; GA, gestation age; VAD, vacuum-assisted delivery; SSCD, second-stage caesarean delivery; BMI, body mass index; FM, family medicine.

† , All data are *n* (%) unless specified otherwise;

‡ , BMI < 18 = Underweight; BMI 18–30 = Healthy; BMI > 30 = Overweight;

§ , Includes all hypertensive disorders of pregnancy;

¶ , This is the primary clinician making the decision as to whether to perform a VAD attempt or SSCD.

Maternal outcomes between the two groups are shown in Table 2. Women who underwent a VAD attempt had a 33% decrease in odds of an adverse maternal outcome (OR 0.67, 95% CI 0.42–1.06; *p* = 0.09). There were statistically significant decreased odds of secondary outcomes of PPH, need for blood transfusion, need for transfer, need for further surgery, and prolonged hospital stay in the VAD attempt group. Major perineal injury only occurred in the VAD attempt group.

**TABLE 2:** Comparison of maternal outcomes between vacuum-assisted delivery attempts and second-stage caesarean delivery.

Characteristics	All†		Intervention type†				*P*	Crude	
			VAD attempt		SSCD			OR‡	95% CI
	*n* (*N* = 567)	%	*n* (*N* = 417)	%	*n* (*N* = 150)	%			
**Outcome of interest**									
Adverse maternal outcome§	99	17	66	16	33	22	0.09	0.67	0.42–1.06
**Maternal outcomes**									
Postpartum haemorrhage¶	43	8	22	5	21	14	**< 0.01**	0.34	0.18–0.64
Need for blood transfusion	21	4	10	2	11	7	**< 0.01**	0.31	0.13–0.75
Major perineal injury††	32	6	32	8	0	0	**< 0.01**	-	-
Need for transfer	13	2	5	1	8	5	**< 0.01**	0.21	0.07–0.67
Need for surgery‡‡	10	2	2	0.5	8	5	**< 0.01**	0.09	0.18–0.41
Uterine rupture	2	0.5	1	< 0.5	1	1	0.22	0.36	0.02–5.76
Maternal death	0	0	0	0	0	0	-	-	-
Prolonged hospital stay§§	30	5	14	3	16	11	**< 0.01**	0.29	0.14–0.61

Note: Bold values indicate statistically significant results (*p* < 0.05).

VAD, vacuum-assisted delivery; SSCD, second-stage caesarean delivery; OR, odds ratio; CI, confidence interval; NVD, normal vaginal delivery; CD, caesarean delivery.

† , All data are *n* (%);

‡ , Bivariate analyses with unadjusted OR of VAD attempts with SSCD as reference;

§ , Composite of all severe adverse maternal outcomes as listed;

¶ , As recorded by the managing clinician (National guidelines: > 500 mL for NVD; > 1000 mL for CD);^6,19^

†† , Grade three or four perineal injuries;

‡‡ , Includes any subsequent surgery including laparotomy; secondary wound closure; hysterectomy;

§§ , Defined as > 5 days.

Perinatal outcomes between the two groups are shown in Table 3. There was no difference for the primary outcome of adverse perinatal outcome composite (OR 0.92, 95% CI 0.48–1.75, *p* = 0.8). There were also no statistically significant differences for secondary outcomes of need for admission, need for transfer, major birth trauma, neonatal seizures, low Apgar score, foetal death after decision to deliver made, and early neonatal death.

**TABLE 3:** Comparison of perinatal outcomes between vacuum-assisted delivery attempt and second-stage caesarean delivery.

Characteristics	All†		Intervention type†				*P*	Crude	
			VAD attempt		SSCD			OR‡	95% CI
	*n* (*N* = 567)	%	*n* (*N* = 417)	%	*n* (*N* = 150)	%			
**Outcome of interest**									
Adverse perinatal outcome§	50	9	36	9	14	9	0.8	0.92	0.48–1.75
**Perinatal outcomes**									
Need for admission	39	7	27	6	12	8	0.53	0.80	0.39–1.61
Need for transfer‡‡	1	< 0.5	0	0	1	1	0.10†	-	-
Major birth trauma¶‡‡	9	2	9	2	0	0	0.07†	-	-
Neonatal seizures‡‡	2	0.5	2	0.5	0	0	0.4†	-	-
Low Apgar score††	23	4	18	4	5	3	0.6	1.31	0.48–3.59
Foetal death after decision to deliver made‡‡	1	< 0.5	0	0	1	1	0.10†	-	-
Early neonatal death	11	2	8	2	3	2	0.48	0.96	0.25–3.66

VAD, vacuum-assisted delivery; SSCD, second-stage caesarean delivery; OR, odds ratio; CI, confidence interval.

† , All data are *n* (%);

‡ , Bivariate analyses with unadjusted OR of VAD attempts with SSCD as reference;

§ , Composite of all severe adverse perinatal outcomes as listed;

¶ , Including subgaleal or subaponeurotic haemorrhage; humerus fracture, clavicle fracture. Excluding minor trauma; cephalhematoma;

†† , 5-min Apgar score < 7;

‡‡ , Two-tailed tests rather than logistic regression analysis.

Table 4 presents the findings of the multivariable logistic regression analysis for the principal study outcomes. Significant covariates from the initial analysis were previous CD, maternal age, parity, number of antenatal care (ANC) visits, gestation age (GA) at booking and delivery, maternal body mass index (BMI), HIV status, maternal hypertensive disorder, level of clinician for decision-making, birth weight, and indication for second stage intervention. After adjusting for these covariates, women who underwent VAD attempt had a 70% decrease in odds of having a PPH (OR 0.30, 95% CI 0.15–0.60) and a 36% decrease in odds of a composite adverse maternal outcome (OR 0.64, 95% CI 0.39–1.05). There were no statistically significant differences for adverse perinatal outcome composite (OR 0.82, 95% CI 0.42–1.60) and low Apgar score (OR 1.22, 95% CI 0.43–3.50).

**TABLE 4:** Multivariable logistic regression analysis of maternal and perinatal outcomes.

Outcomes: VAD attempt vs SSCD	Intervention type			
	SSCD		VAD attempt	
	Adjusted OR	95% CI	Adjusted OR	95% CI
Adverse maternal outcome‡				
Adjusted OR†	1.00	Reference	0.64	0.39–1.05
Adverse perinatal outcome§				
Adjusted OR†	1.00	Reference	0.82	0.42–1.60
Postpartum haemorrhage¶				
Adjusted OR†	1.00	Reference	0.30	**0.15–0.60**
Low Apgar score††				
Adjusted OR†	1.00	Reference	1.22	0.43–3.50

Note: Bold formatting indicates statistically significant findings (95% confidence interval excludes 1.0; equivalent to *p* < 0.05).

OR, odds ratio; CI, confidence interval; VAD, vacuum-assisted delivery; SSCD, second-stage caesarean delivery; BMI, body mass index; CD, caesarean delivery; NVD, normal vaginal delivery; ANC, antenatal care; HIV, human immunodeficiency virus.

† , ORs were adjusted for previous caesarean delivery, maternal age, parity, number of ANC visits, whether early or late booking, gestational age at delivery, maternal BMI, HIV status, maternal hypertensive disorder, level of clinician for decision making, birth weight, and indication for second stage intervention;

‡ , Composite of adverse maternal outcomes as listed in Table 2;

§ , Composite of adverse perinatal outcomes as listed in Table 3;

¶ , As recorded by the managing clinician (National guidelines: > 500 mL for NVD; > 1000 mL for CD).^6,19^

†† , 5-min Apgar score < 7.

## Discussion

This study showed that for women who required intervention in the second stage of labour, there was a reduction in the odds of an adverse maternal outcome after VAD attempt as compared with SSCD. Although the composite adverse maternal outcome did not reach conventional statistical significance, several individual maternal outcomes were significantly less frequent in the VAD attempt group. This reflects the heterogeneity of events within the composite outcome, where outcomes of differing frequency and clinical severity (some favouring VAD attempts and others occurring only in that group) may dilute overall statistical significance despite clinically meaningful differences in key components. Specifically, major perineal injury (which is non-life-threatening) was disproportionately overrepresented as a severe adverse outcome in the VAD attempt group. In contrast, more severe life-threatening complications such as PPH, need for transfer to a tertiary centre and need for further surgery contributed more significantly towards adverse outcomes in the SSCD group. In addition, this study focused on short-term maternal and perinatal outcomes only. This is important to note, as existing literature has shown that CD (particularly when performed in the second stage of labour) is associated with clinically relevant long-term risks, including abnormal placentation, uterine rupture, hysterectomy and increased maternal morbidity in subsequent pregnancies.^3,10,20^ These longer-term outcomes were not assessed in the present study and warrant further investigation, particularly in rural district hospital settings. Lastly, this study included cases of failed VAD and subsequent CD in the VAD attempt group, as well as cases of foetal distress, which differs from similar research conducted in Nigeria (which excluded interventions due to foetal distress).^12^

This study showed that perinatal outcomes of babies delivered after a VAD attempt were equivalent to those following SSCD. These findings are consistent with studies conducted between 2017 and 2020 in tertiary hospitals in Uganda and Nigeria, which similarly reported comparable neonatal outcomes under specialist obstetric care.^12,14^ Although the overall use of VAD has declined over time, these studies were broadly contemporaneous with the current study and reflect clinical practice within the same era. In contrast, a 2017 study from Israel reported poorer neonatal outcomes following SSCD,^13^ highlighting potential contextual differences in practice and case selection.

The sample size of this study was similar to studies conducted in Nigeria and Uganda and larger than studies conducted in Israel and Turkey.^12,13,14,21^ The CD rate of 22% in this study is comparable to studies conducted in Turkey and Nigeria, which reported CD rates of 18% and 20%, respectively.^12,21^ This rate is higher than those observed in some LMICs, which range from 16% to 18%.^7^ However, it is lower than the rates reported in Uganda and Israel of 31% and 29%, respectively.^13,14^ The management of many lower-risk and some higher-risk pregnancies at this rural facility likely contributed to some of the similarities and differences in sample size and CD rate. The VAD attempt rate of 4.8% was higher than similar research from Uganda, Nigeria, Turkey, and other LMICs, ranging from 0.5% to 3.3%.^7,12,14,21^ No accurate rates for South Africa are available, but rates in this study are above the 0.5% – 1% estimates.^8^ The failure rate of 16.9% for VAD attempts was higher than research from Nigeria, Uganda, and global averages of 5.9%, 9.2%, and 13.9%, respectively.^7,12,14^ This, combined with the relatively high VAD attempt rate, suggests the regular use of VAD attempts as a second-stage intervention in this group and the acceptance of failure as not enough of an adverse event in itself to discourage its use.

Baseline clinical characteristics were similar across the two groups and comparable to national averages, suggesting a representative and comparable sample.^20^ Low rates of ANC attendance likely reflect broader structural barriers to healthcare access in rural South Africa, including socioeconomic disadvantage, lower educational attainment, rural residence, and transport limitations, all of which have been shown to reduce ANC utilisation.^4,22,23^ Nulliparity is a recognised risk factor for complications in the second stage of labour, and the proportion of nulliparous women in this cohort is consistent with available literature.^24^ Higher rates of previous CD in the SSCD group are consistent with previous literature identifying this as a risk factor for complications in subsequent pregnancies.^7^ It may have introduced confounding in increasing the odds of adverse outcomes in the SSCD group as well as impacted clinician decision-making between VAD attempt and SSCD. The DDI in this study was shorter for both the VAD attempt and SSCD groups as compared with other literature in Nigeria and Uganda.^12,14^ Shorter DDI in the VAD attempt group likely contributed significantly towards the maternal and perinatal outcomes. This is because indications for intervention in the second stage often involve time-sensitive contributing factors, for example, maternal haemodynamic stability and foetal hypoxia.^8^ The intention-to-treat analysis of the VAD attempt group helps inform the performing clinician that the odds of a shorter expected DDI at this facility remain regardless of whether the VAD attempt was successful or not.

Decision-making in labour is complex and impacted by clinician experience and available resources. Recent research showed that junior doctors in South Africa are not sufficiently equipped with the knowledge and experience to perform instrumental deliveries safely.^25^ As a result, they are more likely to opt for a CD. However, data consistently suggest that maternal and perinatal outcomes from CDs performed by junior doctors in rural settings are poor.^20,26^ Community service medical officers and medical officers constitute a significant portion of the staffing in rural district hospitals.^25^ Their limited knowledge and experience likely contributed towards the differences in decision-making as compared with family medicine registrars and family physicians in this study. The similar decision-making patterns observed among family physicians and family medicine registrars (compared with other clinician categories) suggest a potential stabilising role for family medicine expertise in second-stage obstetric care in rural district hospitals, although this requires further investigation.

Rising CD rates in many countries have not been associated with meaningful improvements in maternal or perinatal outcomes.^8,27^ In fact, they have been associated with adverse outcomes in current and future pregnancies, particularly in rural hospitals.^7,8,20^ In addition, several studies in LMICs and upper-middle-income countries have shown that the use of VAD remains a safe and effective method of intervening in the second stage of labour.^12,13,14,21,27^ This approach does not compromise neonatal outcomes and may even improve maternal outcomes and the overall birthing experience.^14,28,29,30^ Despite this, there is an ongoing aversion to VAD use and decreasing rates of use.^5,8^ This study adds to the literature that the use of VAD is a safe alternative to SSCD, especially in low-resource settings and those with no obstetric specialist support.^8,12,13,14,21^

Despite the value of this study, it is not without limitations. The use of retrospective medical record reviews introduces the possibility of information and selection bias. As an observational cohort study, the findings are also subject to residual confounding, including confounding by indication. In this study, indications for second-stage intervention differed between groups, with foetal distress more commonly prompting VAD attempts and prolonged second stage more frequently resulting in SSCD. These differing clinical contexts are likely to influence both decision-making and outcomes and may have contributed to the observed associations, despite adjustment for indication and other relevant covariates. In addition, this study only assessed short-term outcomes, lacking long-term follow-up data that would provide a more comprehensive understanding of the overall implications. Additionally, variations in baseline characteristics among the two groups, along with data collection being restricted to a single site, further limit the generalisability of these results to broader patient populations and varying clinical contexts. There is a need for a larger multicentre study with a longer follow-up period that explores various aspects of second-stage interventions, including the impact of family physicians and other clinicians. Investigating the complexities of decision-making during the second stage of labour in different healthcare facilities and among a range of clinicians, utilising both qualitative and quantitative research methodologies, could provide valuable insights into the factors contributing to the aversion to VAD. Such understanding may inform strategies to enhance the adoption of VAD practices and promote their reintegration within clinical protocols.

### Recommendations

Further research is needed to better understand factors associated with VAD failure in rural district hospital settings and the subsequent impact on maternal and perinatal outcomes. In addition, future studies should explore the influence of clinician training, experience, and supervision on second-stage obstetric decision-making, particularly in settings without obstetric specialist support.

Based on the findings of this study, the use of VAD as a second-stage intervention appears safe in appropriately selected cases in rural district hospitals. Training in VAD skills should therefore be prioritised in these settings to support timely decision-making and reduce unnecessary SSCD. Vacuum-assisted delivery competency should form a core component of clinical training programmes in rural hospitals, particularly where family physicians and family medicine registrars contribute to second-stage obstetric care.

## Conclusion

Vacuum-assisted delivery attempts, performed in a rural district hospital setting in South Africa by medical doctors with no obstetric specialist support, are safe alternatives to SSCD with improved maternal and equivalent perinatal short-term outcomes. Considering the adverse long-term outcomes of CD, including the risk for future pregnancies, these findings support safe use of VAD as an alternative second-stage intervention in appropriately selected cases within rural district hospitals.
